# Adapting evidence-based clinical practice guidelines for people with attention deficit hyperactivity disorder in Saudi Arabia: process and outputs of a national initiative

**DOI:** 10.1186/s13034-020-00351-5

**Published:** 2021-02-08

**Authors:** Fahad A. Bashiri, Turki H. Albatti, Muddathir H. Hamad, Haya F. Al-Joudi, Hadeel F. Daghash, Saleh M. Al-Salehi, Jeremy L. Varnham, Fatimah Alhaidar, Omar Almodayfer, Abdulkarim Alhossein, Hesham Aldhalaan, Yasser A. Ad-Dab’bagh, Nouf Al Backer, Waleed Altwaijri, Khalid Alburikan, Maysaa W. Buraik, Mohammad Ghaziuddin, Michael J. Nester, Hayfaa A. Wahabi, Samia Alhabib, Amr A. Jamal, Yasser S. Amer

**Affiliations:** 1 Pediatrics Department, Pediatric Neurology Division, College of Medicine and King Saud University Medical City, King Saud University, Riyadh, Saudi Arabia; 2 Saudi Pediatric Neurology Society, Riyadh, Saudi Arabia; 3 Psychiatry Department, Child Psychiatry Unit and King Saud University Medical City, King Saud University, Riyadh, Saudi Arabia; 4 Saudi ADHD Society, Riyadh, Saudi Arabia; 5 Ministry of Education, Abdullatif Alfozan Autism Center, Al Khobar, Saudi Arabia; 6 Department of Neurosciences, King Faisal Specialist Hospital & Research Centre, Riyadh, Saudi Arabia; 7 Ada’a Program, Assistant Deputyship for Hospital Services, Ministry of Health, Riyadh, Saudi Arabia; 8 King Abdullah Bin Abdulaziz University Hospital, Princess Nourah Bint Abdulrahman University, Riyadh, Saudi Arabia; 9 King Abdulaziz Medical City, Riyadh, Saudi Arabia; 10 Department of Special Education, King Saud University, Riyadh, Saudi Arabia; 11 Department of Neurosciences, Center for Autism Research, King Faisal Specialist Hospital & Research Centre, Riyadh, Saudi Arabia; 12 Department of Mental Health, Neuroscience Center, King Faisal Specialist Hospital-Dammam (KFSH-D), Dammam, Saudi Arabia; 13 Research Center, King Khalid Medical City (RC-KKMC), Dammam, Saudi Arabia; 14 Department of Pediatrics, Developmental-Behavioral Pediatrics Division, College of Medicine and King Saud University Medical City, King Saud University, Riyadh, Saudi Arabia; 15 Saudi Pharmaceutical Society, Riyadh, Saudi Arabia; 16 Neuroscience Institute, Psychiatry Division, Johns Hopkins Aramco Healthcare, Dhahran, Saudi Arabia; 17 Department of Psychiatry, University of Michigan, Ann Arbor, USA; 18 Child and Adolescent Services, Ann Arbor, USA; 19 Research Chair for Evidence-Based Health Care and Knowledge Translation, King Saud University, Riyadh, Saudi Arabia; 20 National Centre for Evidence-Based Health Practice, Saudi Health Council, Riyadh, Saudi Arabia; 21 Family and Community Medicine Department, College of Medicine and King Saud University Medical City, King Saud University, Riyadh, Saudi Arabia; 22 National Centre for Health Information, Saudi Health Council, Riyadh, Saudi Arabia; 23 CPG Unit, Quality Management Department, King Saud University Medical City, Riyadh, Saudi Arabia; 24 Pediatrics Department, King Saud University Medical City, Riyadh, Saudi Arabia; 25 Alexandria Center for Evidence-Based Clinical Practice Guidelines, Alexandria University, Alexandria, Egypt

**Keywords:** Practice guideline, Adaptation, Evidence-based medicine, ADHD, Attention deficit hyperactivity disorder, Mental health, Saudi Arabia, Eastern mediterranean region

## Abstract

**Background:**

We recently adapted the published National Institute for Health and Care Excellence (NICE) Attention deficit hyperactivity disorder (ADHD) diagnosis and management guideline to the Saudi Arabian context. It has been postulated that adaptation of evidence-based clinical practice guidelines to the local healthcare context rather than de-novo development will improve their adoption and implementation without imposing a significant burden on resources. The objective of this paper is to describe the adaptation process methodology utilized for the generation of the first national guideline for management of people with ADHD in Saudi Arabia.

**Methods:**

We used the KSU-Modified-ADAPTE methodology for the guideline adaptation process. We describe the full process in detail including the three phases of set-up, adaptation, and finalization. The process was conducted by a multidisciplinary guideline adaptation group in addition to an external review for the clinical content and methodology.

**Results:**

The group adapted ten main categories of recommendations from one source CPG (NICE). The recommendations include: (i) service organisation and training, (ii) recognition, identification and referral, (iii) diagnosis, (iv) support, (v) managing ADHD, (vi) dietary advice, (vii) medication, (viii) maintenance and monitoring, (ix) adherence to treatment, and (x) review of medication and discontinuation. Several implementation tools were compiled and developed to enhance implementability including a clinical algorithm, quality measures, coding system, medication tables, translations, patient information, and online resources.

**Conclusions:**

The finalized clinical practice guideline provides healthcare providers with applicable evidence-based guidance for the management of people with ADHD in Saudi Arabia. The project also demonstrated the effectiveness of KSU-Modified-ADAPTE, and emphasized the value of a collaborative clinical and methodological expert group for adaptation of national guidelines.

## Contributions to the literature

- Adaptation of guidelines is a valid alternative to de novo development for generation of evidence-based guidelines.
- The ‘King Saud University (KSU)-Modified-ADAPTE’, as a formal methodology for guideline adaptation, is less resource-intensive than de-novo development without losing the methodological rigor.
- Balanced clinical and methodological expertise in the guideline group is essential for the success of similar projects.
- We describe the process and outputs of a comprehensive national guideline adaptation initiative with multidisciplinary contributions for management of people with ADHD.
- These findings contribute to the work to enhance adaptation or customization of clinical practice guidelines and highlight implementability issues for ADHD.

## Background

Attention deficit hyperactivity disorder (ADHD) is one of the most common neurodevelopmental disorders that affects cognitive, emotional, social, academic, and occupational functioning [1]. It is classified into three main presentations: predominantly inattentive, predominantly hyperactive/impulsive and combined presentation [2]. Although classified as a childhood-onset disorder, it may continue into adolescence and adult life. The worldwide prevalence of ADHD is estimated to be around 5–7% of children and adults. A number of regional studies have been conducted into the prevalence of ADHD in Saudi Arabia [3–6], but as yet without definitive national significance. It is recognized to have a significant burden if under-recognized and untreated. Internationally, ADHD is managed in various shared-care models between primary and secondary care that best suit each country’s individual resources, culture, and nature of practice. The diagnosis of ADHD is based on the diagnostic criteria in the Diagnostic and Statistical Manual of Mental Disorders – 5th Edition (DSM-5) [2] or the International Statistical Classification of Diseases and Related Health Problems, 10th Revision (ICD-10) (hyperkinetic disorder) [7]. Although in Saudi Arabia, the official coding system that has been adopted is the Australian revision (ICD-10-AM), whose terminology differs slightly (disturbance of activity and attention, hyperkinetic conduct disorder, other hyperkinetic disorders), the term ADHD is widely recognized [7].

There were no standardized clinical guidelines for ADHD management in Saudi Arabia. There is, however, a large volume of internationally published CPGs for ADHD that may create a dilemma for relevant healthcare providers and clinicians who care for people with ADHD in Saudi Arabia during the processes of sharing healthcare decisions and care provision. Furthermore, although some initiatives have targeted the management of ADHD in primary care, they are in their infancy; ADHD is mainly diagnosed and treated in tertiary care and the private sector and managed in a variety of settings, sometimes inappropriately or ineffectively. This results in significant variability in clinical practice, and suboptimal quality of care [8–10].

As part of its strategy to improve access to care for people affected by ADHD in Saudi Arabia, the Saudi ADHD Society formed a multidisciplinary team to remedy this situation. The resulting clinical practice guideline (CPG) was adapted from the National institute for Health and Care Excellence (NICE) guideline entitled, Attention deficit hyperactivity disorder: diagnosis and management (NG87) [11], to improve recognition, diagnosis and quality of care for patients with ADHD.

Clinical Practice Guidelines (CPGs) are defined as ‘statements that include recommendations intended to optimize patient care, which are informed by a systematic review of evidence and an assessment of the benefit and harm of alternative care options [12]. CPGs have been identified as one of the main tools for improving evidence-based healthcare quality and safety [12, 13].

Adaptation of CPGs is a valid and efficient alternative to de novo development of CPGs especially in resource-limited healthcare settings. It was proposed to avoid duplication of efforts, to use the available resources in a cost-effective manner, and to encourage trans-contextual customization of the CPG prepared for different economic and healthcare settings reflecting the local context and system [12–16].

Given that there were no published CPGs for ADHD management in Saudi Arabia, the presented evidence-based CPG is proposed as a National CPG using an evidence-based and formal CPG adaptation methodology. The aim of this study was to adapt the international clinical practice guidelines’ recommendations for people with ADHD to fit the healthcare setting in the Saudi Arabian context including primary, secondary, and tertiary care settings.

## Methods

### Guideline adaptation methodology

**Fig. 1:**
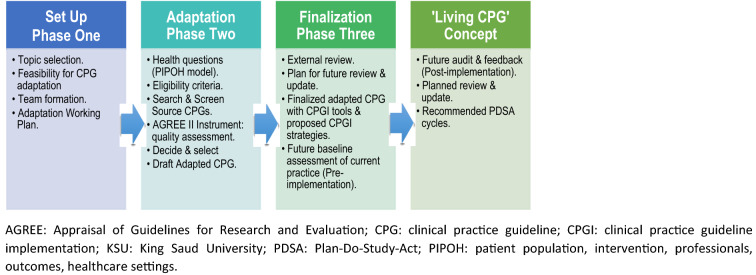
Summary of the ‘KSU Modified ADAPTE’ process for CPG adaptation

We utilised the ‘King Saud University (KSU)-Modified-ADAPTE’ [12] adaptation methodology, a natural evolution of two earlier formal adaptation methodologies for CPGs, the original ADAPTE and ‘Adapted ADAPTE’ methods [15–17], which consists of three phases and 24 steps with modifications in the steps and tools to suit the local general healthcare setting in Saudi Arabia [12, 16]. Figure 1 provides a simplified flowchart of our methods [12].

The two main reporting standards for CPGs recommended by the EQUATOR (Enhancing the QUAlity and Transparency Of health Research) Network are the Appraisal of Guidelines for Research and Evaluation (AGREE) II reporting checklist and the Reporting Items for practice Guidelines in HealThcare (RIGHT) statement [18–20]. Despite the fact that these tools were designed for de-novo developed CPGs, rather than adapted CPGs, we will report our adapted CPG for ADHD using the AGREE II reporting checklist after applying specific explanations to the items of the checklist relevant to our CPG adaptation process in contrast to a CPG development process (Additional file 1). Furthermore, there is an ongoing research project for developing an extension of the RIGHT statement for the reporting of ‘adapted CPGs’ (RIGHT-Ad@pt Checklist) [21].

### Phase one (set up)

In phase one, Attention deficit hyperactivity disorder (ADHD) was identified by the Saudi ADHD Society as the health topic for this CPG adaptation project. An initial exploratory search regarding ADHD CPGs was conducted to identify whether there were existing CPGs related to this topic. The guideline adaptation working group (GAG) was formulated at the outset to include a child psychiatrist, two pediatric neurologists, a developmental pediatrician, a clinical neuropsychologist, a clinical pharmacist, a general pediatrician and CPG expert methodologist, a project manager, and a patient advocate. Participation of the patient advocate in the GAG and all of its meetings was intended to capture the patients’ or public’s views and preferences in addition to the support and insight from the networks and resources of the Saudi ADHD Society. The results of the preliminary search for ADHD Source CPGs encouraged us to proceed and officially launch this CPG adaptation project with a national scope. Capacity building sessions were conducted by the CPG methodologist for the rest of the GAG on concepts of evidence-based healthcare including the CPG adaptation process methodology and its associated toolkit [12, 14, 17].

The target patient population for the adapted CPG is children and adults suspected of having or diagnosed with ADHD. The identified target intended users include physicians, clinical psychologists, other behavioral health clinicians, nurses, occupational therapists, pharmacists, social workers, dieticians, medical students, and health sciences students.

The healthcare settings include primary, secondary and tertiary care dealing with assessment, treatment and management of ADHD in Saudi Arabia.

### Phase two (adaptation)

In phase two, we identified specific health questions using the PIPOH model, relevant inclusion and exclusion criteria, and a full search strategy including a list of keywords. The elements of the PIPOH model include the target patient population (P), intervention(s) (I), professionals and clinical specialties (P), outcomes (O), and healthcare setting or context (H) that were reported earlier [4, 12]. We searched eight bibliographic and CPG databases in addition to online libraries of relevant professional societies. Eligible Source CPGs for ADHD were then critically appraised using the AGREE II Instrument [22]. AGREE II is a valid and reliable instrument with 23 items organized into six domains and is considered the gold standard for quality assessment of CPGs [22]. A cut-off point of 60% for each AGREE II standardized domain score was agreed upon by the members of the GAG [1].

Based on the results of the AGREE II appraisal [1] and in-depth content review of the source CPG from NICE, there was a consensus among the members of the GAG that the recommendations were clear and were based on the most relevant scientific evidence, applicable to the local context, and acceptable to people with ADHD.

We decided not to conduct further assessment of the certainty of the body of evidence and the strength of recommendations and relied on the high standardized domain score of domain 3 (rigour of development) of the AGREE II appraisal and the evidence-base of the NICE source CPG based on its provided Grading of Recommendations: Assessment, Development, and Evaluation (GRADE) evidence profiles [1].

Moreover, the GAG identified, revised, and discussed all the recommendation statements through successive focus group discussions against the local and national healthcare system in Saudi Arabia. Drafting the first version of the adapted CPG was the last step of this phase.

### Phase three (finalization)

In phase three, the first draft of the adapted CPG full document was finalized including assessing the recommendations for acceptability and applicability in the local Saudi Arabian healthcare settings. This adapted CPG draft was then disseminated to a selected national panel of external reviewers of specialized healthcare providers, topic experts, and methodologists from relevant healthcare sectors. The feedback of reviewers was revised and discussed within the GAG and was reflected in the final version of the adapted CPG. A set of CPG implementation (CPGI) tools was included in the final CPG full document.

## Results

The overall duration of this CPG adaptation project was two years and five months from 4^th^ of January 2017 till 30th of May 2019. Seven meetings were conducted for planning, reviewing, and focus group discussions including two training sessions with ongoing hands-on advisory on the CPG appraisal and adaptation tools.

This work marks the first national CPG adaptation project for the management of people with ADHD using the ‘KSU-Modified-ADAPTE’.

### Phase one (set up)

The aforementioned GAG was formulated in January 2017 as a multidisciplinary group with expertise in ADHD (TA, FB, MH, HA, SA, HD) and evidence-based CPGs (YA). ADHD was selected as a high priority health topic with clear practice variation and lack of national CPGs for its management. The necessary resources and skills were identified and allocated. All of the GAG members signed declaration of conflicts of interest statements.

The feasibility of the CPG adaptation process was confirmed by conducting a preliminary search for published CPGs. The working plan was drafted and discussed at the outset using the relevant CPG adaptation working plan template from the KSU-Modified-ADAPTE (Appendix, Table 3) [12].

### Phase two (adaptation)

For the first and second phases, a systematic review for the recently published ADHD Source CPGs was conducted and published in a separate report, which included the PIPOH model, eligibility criteria, results of the search and screen for Source CPGs, in addition to the results of the ratings and commentary of the AGREE II appraisal [1].

Six source ADHD CPGs were reviewed and critically appraised including those developed by the American Academy of Pediatrics, Canadian ADHD Resource Alliance, National Health and Medical Research Council, National Institute for Health and Care Excellence (NICE), Singapore Ministry of Health, and University of Michigan Health System [1].

The NICE CPG was superior in all of the six standardized domain scores of the AGREE II Instrument and it addressed all care options for ADHD across the lifespan. The AGREE II ratings of the NICE CPG were 100% (domain 1: scope and purpose), 96% (domain 2: stakeholder involvement), 93% (domain 3: rigour of development), 89% (domain 4: clarity and presentation), 92% (domain 5: applicability), 92% (domain 6: editorial independence), and 100% (overall assessment 1) [1].

Afterwards, we assessed the currency of the NICE Source CPG to ensure the validity and currency of its recommendations and their evidence-base using the related assessment of the CPG currency from the KSU-Modified-ADAPTE (Appendix, Table 4) [11, 12].

The GAG reviewed and discussed the AGREE II assessment standardized domain scores and decided to adopt all of the recommendations of the NICE CPG. Relevant customization of the recommendations was conducted after several focus group discussions of facilitators and barriers to CPGI especially regarding variable health systems, medications, or healthcare provider positions.

We have followed the same format or presentation of recommendation statements developed by NICE that relied on the ‘wording’ of each recommendation rather than highlighting a quality of evidence and grade of recommendation like other CPG developers may opt for. The rationale for this format has been clearly stated in the NICE website: https://www.nice.org.uk/about/what-we-do/our-programmes/nice-guidance/nice-guidelines/making-decisions-using-nice-guidelines in addition to further explanation in, Chapter 9: writing the guideline of, ‘Developing NICE guidelines: the manual’: https://www.nice.org.uk/process/pmg20/chapter/writing-the-guideline

**Fig. 2:**
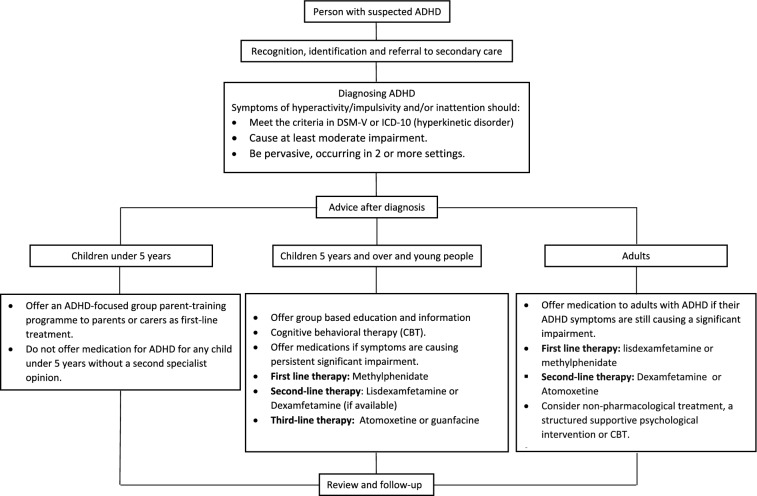
Clinical algorithm for management of ADHD

The GAG decided to adopt the CPGI tools provided by the NICE Source CPG, i.e. baseline assessment tool and quality standards. Additional CPGI tools were included by the GAG based on and relevant to the adapted ADHD recommendations including: (i) two medication tables; one for treatment of children and young people and the other for treatment of adults with ADHD (a summary medication table has been provided in this article), (ii) a clinical algorithm for management of ADHD (Fig. 2), (iii) the set of related ICD-10-AM codes that were adopted by the National Health Information Center, Saudi Health Council [7] in addition to the ICD-11 codes [23], and (iv) links to patient educational information and resources on the Society’s official website. A mobile-friendly web-based version of the CPG was also developed.

### Phase three (finalization)

Thirteen members participated as the external review panel from the target audience of the CPG based on their expertise in caring for people with ADHD (FA, OA, AA, HA, YAA, NA, WA, KA, AJ, and MB) and in methodologies of evidence-based CPGs (HAA and SA) in addition to their representation of multiple relevant healthcare sectors in Saudi Arabia. Two international experts with local experience were invited to contribute to the external review of the clinical content as well (MG and MJN).

The external review comments were compiled using a template [12], revised, discussed, and incorporated in the recommendations and implementation tools of the finalized adapted CPG full document.

The customization or adaptation of recommendations was conducted with regards to the differences in the health systems and delivery of healthcare services especially for people with ADHD between the United Kingdom (UK) and the Kingdom of Saudi Arabia. The similarities in the health systems in both countries being nationalized healthcare systems where the government provides the majority of healthcare services, in addition to the similarity of income levels, facilitated the process of adaptation of recommendations to the local context [24]. Furthermore, the recommended medications were revised against those currently approved by the Saudi Food and Drug Authority (Saudi FDA), and those available on a restricted basis through specific hospitals. No formal cost-analyses or Health Technology Assessment(s) were conducted as part of this project.

Health benefits, side effects, and risks were evaluated in the Source CPG (NICE) as part of the AGREE II assessment [1] and were further revised and discussed during the adaptation or customization of the recommendations to the local context.

The values and preferences of the target patient population was considered and discussed throughout the CPG adaptation process through the input of the patient advocate. Moreover, reports from the patient and public encounters during related services were provided by the society.

As a part of quality assurance, the finalized adapted CPG from the Saudi ADHD Society was then critically reviewed and endorsed by the Saudi Health Council as well as five national professional societies: the Saudi Pediatric Neurology Society, the Saudi Pediatric Association, the Saudi Pharmaceutical Society, the Saudi Psychiatric Association, and the Saudi Society of Professional Psychology. The adapted CPG included recommendation statements organized into ten sections including: (i) Service organisation and training, (ii) Recognition, identification and referral, (iii) Diagnosis, (iv) Support, (v) Managing ADHD, (vi) Dietary advice, (vii) Medication, (viii) Maintenance and monitoring, (ix) Adherence to treatment, and (x) Review of medication and discontinuation.

The Saudi ADHD Society contacted NICE, the Source CPG developer, and finalized an official end user license agreement in line with the original NICE terms and conditions and the NICE UK Open Content license.

A summary of the key recommendations is provided in Table 1 and the full CPG document is made available, in addition to the translation into the Arabic language [25], on a user-friendly and accessible microsite of the official website of the Saudi ADHD Society: https://cpg.adhd.org.sa/.

**Table 1:** Summary of the Key recommendations in the adapted clinical practice guideline for the management of children and adults with ADHD

*Recognition*
There are certain groups may have increased prevalence of ADHD compared to the general population like: People born preterm Looked-after children (e.g. those living in care homes such as orphanages or juvenile detention facilities) People with oppositional, conduct disorders or mood disorders People with neurodevelopmental disorders (for example autistic spectrum disorders, tics, intellectual disability, and specific learning difficulties) People with a close family member diagnosed with ADHD People with epilepsy Adults with a mental health condition People with a history of substance misuse People with acquired brain injury
*Identification and referral*
We recommend that universal screening for ADHD should not be undertaken in nursery, primary and secondary schools. When a child or young person with disordered conduct and suspected ADHD is referred to a school’s special education teacher or consulting teacher, in addition to helping the child with its behavior, he/she should inform the parents about local specialized programmes (e.g. General Pediatric clinics, Developmental and Behavioral Clinics, etc.)
*Diagnosis*
The diagnosis of ADHD is based on the diagnostic criteria in the Diagnostic and Statistical Manual of Mental Disorders – 5th Edition (DSM-5) or the International Statistical Classification of Diseases and Related Health Problems, 10th Revision (ICD-10) (hyperkinetic disorder). It should be made by a specialist psychiatrist, specialized pediatrician, an appropriately trained family physician or other appropriately qualified healthcare professional with training and expertise in the diagnosis of ADHD after a full clinical, psychosocial, developmental and psychiatric assessment and use of standard rating scales like Conners' rating and Vanderbilt scales. *Note: Currently, the national adopted system is ICD-10-AM*
*Management*
Proper management of patients with ADHD includes early recognition and referral to specialized service and a comprehensive shared treatment plan with the patients and their families. It requires a multidisciplinary approach that involves behavioral therapy, school intervention, parents’ education, and pharmacotherapy. The goals of treatment are to reduce functional impairment and to improve the quality of life *Children under 5 years* ADHD-focused group parent-training programme is the first-line treatment for children under 5 years of age. Medications should not be offered for any child under 5 years without a second opinion from an ADHD service with expertise in managing ADHD in young children *Children aged 5 years and over and young people* Group-based education and information on the causes and impact of ADHD should be given to parents and carers of all children aged 5 years and over and young people with ADHD. A course of Cognitive Behavioral Therapy (CBT) should be considered for those who have benefited from medication but still having a significant impairment in at least one domain Medications should be offered for patients with a persistent significant impairment The diagnosis should be confirmed before offering any medications and the patient should have full assessment for the presence of coexisting medical, mental or neurodevelopmental conditions *First-line therapy* Methylphenidate (either short or long-acting) should be offered as the first-line pharmacological treatment for children aged 5 years and over and young people with ADHD *Second-line therapy* Switching to Lisdexamfetamine should be considered for children who have had a 6-week trial of Methylphenidate Dexamphetamine should be considered for children aged 5 years and over and young people whose ADHD symptoms are responding to Lisdexamfetamine but who cannot tolerate the longer effect profile *Third-line therapy* Atomoxetine or Guanfacine should be offered to children aged 5 years and over and young people if they cannot tolerate methylphenidate or Lisdexamfetamine or their symptoms have not responded to separate 6-week trials of Lisdexamfetamine and Methylphenidate, having considered alternative preparations and adequate doses *Adults* Medications to adults with ADHD should be offered if their ADHD symptoms are still causing significant impairment in at least one domain after environmental modifications have been implemented and reviewed Non-pharmacological treatment should be considered for adults who have difficulty adhering to medications or those who found medication to be ineffective or cannot tolerate it A structured, supportive psychological intervention should be offered for adults with ADHD. Treatment may involve elements of or a full course of CBT *First-line therapy* Lisdexamfetamine or Methylphenidates should be offered as first-line pharmacological treatment Switching to Methylphenidate or Lisdexamfetamine should be considered for adults who have had a 6-week trial of Lisdexamfetamine or methylphenidates at an adequate dose but have not derived enough benefit *Second-line therapy* Dexamfetamine should be considered for adults whose ADHD symptoms are responding to Lisdexamfetamine but who cannot tolerate the longer effect profile Atomoxetine should be offered to adults if they cannot tolerate Lisdexamfetamine or Methylphenidate or their symptoms have not responded to separate 6-week trials of Lisdexamfetamine and Methylphenidate, having considered alternative preparations and adequate doses *Further medication choices* The following medications should not be offered without advice from a tertiary ADHD service: (i) Guanfacine for adults, (ii) Clonidine for children with ADHD and sleep disturbance, rages or tics and (iii) atypical antipsychotics in addition to stimulants for people with ADHD and coexisting pervasive aggression, rages or irritability We recommend offering the same medication choices to people with ADHD and anxiety disorder, tic disorder or autism spectrum disorder as other people with ADHD. We also recommend stopping any medication for children aged 5 years and over, young people and adults with ADHD experiencing an acute psychotic or manic episode. Restarting or starting new ADHD medication after the episode has resolved should be considered
*Maintenance and monitoring*
We recommend the followings: Monitor effectiveness of medication and adverse effects Regular measurement of weight, height and BMI for people taking medication for ADHD Monitor heart rate and blood pressure and compare with the normal range for age before and after each dose change and every 6 months Do not offer routine blood tests or ECGs to people taking medication for ADHD unless there is a clinical indication If a person taking guanfacine has sustained orthostatic hypotension or fainting episodes, reduce their dose or switch to another ADHD medication If a person taking stimulants develops tics, think about whether the tics are related to the stimulant (tics naturally wax and wane) and the impairment associated with the tics outweighs the benefits of ADHD treatment. If tics are stimulant related, reduce the stimulant dose, or consider changing to guanfacine (in children aged 5 years and over and young people only), Atomoxetine, Clonidine or stopping medication Monitor young people and adults with ADHD for sexual dysfunction (that is, erectile and ejaculatory dysfunction) as potential adverse effects of Atomoxetine If a person with ADHD develops new seizures or a worsening of existing seizures, review their ADHD medication and stop any medication that might be contributing to the seizures. After investigation, cautiously reintroduce ADHD medication if it is unlikely to be the cause of the seizures Monitor the behavioral response to medication, and if behavior worsens adjust medication and review the diagnosis
*Dietary advice*
A balanced diet, good nutrition and regular exercise for patients with ADHD is advised. Elimination of artificial coloring and additives from the diet should not be advised. A referral to dietitian should be offered if a relationship was found between behaviors and specific food or drinks

For the complete set of recommendations of the adapted guideline, please refer to the official website: https://cpg.adhd.org.sa/recommendations/

### Plan for scheduled review and update

The GAG recommended for the next review of this adapted CPG to be after four years from its publication (2020) which should be on (2024) after checking for updates in the Source CPG, consultation of expert opinion on any suggested updates needed according to the newest evidence and recommendations published in this area in addition to the implementation and evaluation results at relevant healthcare organizations in the Kingdom of Saudi Arabia. The Checklist for the Reporting of Updated Guidelines (CheckUp) is recommended by the EQUATOR network to report the updating of CPGs [26].

### Implementation considerations and tools

A full set of CPGI tools was an integral component of the adapted CPG full document (Fig. 2, Tables 1, 2). Several CPGI interventions or strategies were highlighted and proposed to promote future multi-faceted CPGI including; (i) leadership engagement and commitment, (ii) dissemination, (iii) clinical and quality champions, (iv) training and education, (v) audit and feedback, (vi) networking with existing projects in the organizations (e.g. performance improvement, accreditation, educational, and scientific activities), and (vi) patients as champions for change [27–29]. Social media, online audio-visual and educational material are key components for launching the dissemination and implementation of this national CPG.

**Table 2:** Summary of medications prescribed for the management of children and adults with ADHD

Medications	Dose	Comments
***Methylphenidate*** ***Immediate-release*** (RITALIN)	***Child 4–5 years***: ***Starting dose***: 2.5 mg twice daily, PO, increased in steps of 2.5 mg daily if required, at weekly intervals ***Maximum dose***: 1.4 mg/kg daily in 2–3 divided doses ***Child 6–17 years***: ***Starting dose: ***5 mg 1–2 times a day, PO, increased in steps of 5–10 mg daily if required, at weekly intervals increased if necessary up to 60 mg daily in 2–3 divided doses, increased if necessary up to 2.1 mg/kg daily in 2–3 divided doses ***Maximum dose***: 60 mg daily in 2–3 doses, higher dose (up to a maximum of 90 mg daily) under the direction of a specialist	***Cautions***: Agitation, anxiety, drug dependence, epilepsy (discontinue if increased seizure frequency), family history of Tourette syndrome, susceptibility to angle-closure glaucoma, and tics ***Contraindications***: Anorexia nervosa, arrhythmia, cardiomyopathy, cardiovascular disease, cerebrovascular disorders, heart failure, hyperthyroidism, phaeochromocytoma, psychosis, severe depression, severe hypertension, structural cardiac abnormalities, suicidal ideation, uncontrolled bipolar disorde, and vasculitis ***Common side effects***: Alopecia, anxiety, decreased appetite, arrhythmias. Arthralgia, Abnormal behaviour, cough, depression, diarrhoea, dizziness, drowsiness, dry mouth, fever, gastrointestinal discomfort, growth retardation, headaches, hypertension, laryngeal pain, altered mood, movement disorders, nasopharyngitis, nausea, palpitations, sleep disorders, vomiting, and decreased weight
***Modified-release*** CONCERTA ® XL	***Starting dose***: 18 mg once daily to be taken in the morning, increased in steps of 18 mg every week adjusted according to response increased if necessary up to 2.1 mg/kg daily ***Maximum dose: ***54 mg once daily ***Dose equivalence and conversion*** Total daily dose of 15 mg of standard-release formulation is considered equivalent to Concerta ® XL 18 mg once daily	
***Lisdexamfetamine***	***Child 6–17 years***: ***Starting dose***: 30 mg once daily, PO, alternatively initially 20 mg once daily, increased in steps of 10–20 mg every week if required, dose to be taken in the morning, discontinue if response insufficient after 1 month; ***Maximum dose***: 70 mg per day	***Cautions***: Bipolar disorder, history of cardiovascular disease, history of substance abuse, may lower seizure threshold (discontinue if seizures occur), psychotic disorders, susceptibility to angle-closure glaucoma, tics, and Tourette syndrome ***Contraindications***: Advanced arteriosclerosis, agitated states, hyperthyroidism, moderate to severe hypertension, and symptomatic cardiovascular disease ***Common side effects***: Upper abdominal pain, anxiety, decreased appetite, abnormal behaviour, constipation, depression, diarrhoea, dizziness, drowsiness, dry mouth, dyspnoea, fatigue, feeling jittery, fever, headache, insomnia, mood altered, nausea, palpitations, psychiatric disorders, skin reactions, tachycardia, tremor, vomiting, and decreased weight
***Dexamfetamine***	***Child 6–17 years***: ***Starting dose: ***2.5 mg 2–3 times a day,PO, increased in steps of 5 mg once weekly if required ***Maximum dose***: 1 mg/kg daily, up to 20 mg daily (40 mg daily has been required in some children). maintenance dose to be given in 2–4 divided doses	***Cautions***: Anorexia, bipolar disorder, history of epilepsy (discontinue if seizures occur), mild hypertension, psychosis, susceptibility to angle-closure glaucoma, tics, and Tourette syndrome ***Contraindications***: Agitated states, cardiovascular disease, history of drug abuse, hyperexcitability, hyperthyroidism, moderate hypertension, severe hypertension, and structural cardiac abnormalities ***Common side effects***: Abdominal pain, anxiety, decreased appetite, arrhythmias, arthralgia, abnormal behaviour, depression, dry mouth, headache, altered mood, movement disorders, muscle cramps, nausea, palpitations, poor weight gain, sleep disorders, vertigo, vomiting, and decreased weight
***Atomoxetine***	***Child 6–17 years (body-weight up to 70 kg)***: ***Starting dose***: 0.5 mg/kg daily, PO, for 7 days, dose is increased according to response; maintenance 1.2 mg/kg daily, total daily dose may be given either as a single dose in the morning or in 2 divided doses with last dose no later than early evening, ***Maximum dose***: 1.8 mg/kg/day or 120 mg per day (high daily doses to be given under the direction of a specialist) ***Child 6–17 years (body-weight 70 kg and above)***: ***Starting dose***: 40 mg daily for 7 days, dose is increased according to response; maintenance 80 mg daily, total daily dose may be given either as a single dose in the morning or in 2 divided doses with last dose no later than early evening, high daily doses to be given under the direction of a specialist ***Maximum dose***: 120 mg per day	***Cautions***: Aggressive behaviour, cardiovascular disease, cerebrovascular disease, emotional lability, history of seizures, hostility, hypertension, mania, psychosis, QT interval prolongation, structural cardiac abnormalities, susceptibility to angle-closure glaucoma and tachycardia ***Contraindications*** Phaeochromocytoma, severe cardiovascular disease. Severe cerebrovascular disease ***Common side effect***s: Anxiety, decreased appetite, asthenia, chest pain, constipation, depression, dizziness, drowsiness, gastrointestinal discomfort, headaches, insomnia, altered mood, mydriasis, nausea, skin reactions, tic, vomiting and decreased weight
***Guanfacine***	Child 6–12 years (body-weight 25 kg and above): ***Starting dose: ***1 mg once daily; PO, adjusted in steps of 1 mg every week if necessary and if tolerated; maintenance 0.05–0.12 mg/kg once daily ***Maximum dose***: 4 mg per day Child 13–17 (body weight 41.5–49.4 kg): maximum dose 5 mg, Child 13–17 (body weight 49.5–58.4 kg): maximum dose 6 mg Child 13–17 (body weight 58.4 kg and above): maximum dose 7 mg	***Cautions***: Bradycardia (risk of torsade de pointes), heart block (risk of torsade de pointes), history of cardiovascular disease, history of QT-interval prolongation, and hypokalaemia (risk of torsade de pointes) ***Common side effects***: Anxiety, decreased appetite, arrhythmias, asthenia, constipation, depression, diarrhoea, dizziness, drowsiness, dry mouth, gastrointestinal discomfort, headache, hypotension, mood altered, nausea, skin reactions, sleep disorders, urinary disorders, vomiting, and increased weight
***Clonidine***	Child ≥ 6 year and adolescent): ***Immediate-release product (PO)***: ***Starting dose***: 0.05 mg at bed time; if needed, increase by 0.05 mg every 3–7 days ***Maximum dose***: 0.4 mg/24 h in 3–4 divided doses ***Extended-release product (PO)***: ***Starting dose***: 0.1 mg at bed time; if needed increase by 0.1 mg every 7 days BID ***Maximum dose***: 0.4 mg/24 h	***Cautions***: Cerebrovascular disease, constipation, heart failure, history of depression, mild to moderate bradyarrhythmia, polyneuropathy, Raynaud’s syndrome or other occlusive peripheral vascular disease, sleep disturbance, rages or tics ***Contraindications*** Severe bradyarrhythmia secondary to second—or third-degree AV block or sick sinus syndrome ***Common side effects***: Constipation, depression, dizziness, dry mouth, fatigue, headache, nausea, postural hypotension, salivary gland pain, sedation, sexual dysfunction, sleep disorders, and vomiting

For the complete Medication tables, please refer to the official link: https://cpg.adhd.org.sa/implementation-tools-considerations/medication-tables/

The GAG recommends using this adapted CPG as a core tool within regular Plan-Do-Study-Act (PDSA) healthcare quality improvement cycles to support and promote quality and safety of healthcare services and best practice for people with ADHD.

### Facilitators and barriers to implementation

Several potential facilitators and barriers to implementation were identified during the CPG adaptation process.

Facilitators include the relevant national strategies, committees, initiatives, and new healthcare services that are expected as a part of the new model of care, to support implementation. Contribution of representatives of multiple local healthcare sectors are designed to facilitate early dissemination and implementation. Furthermore, leadership engagement and support from the organizing society and from the contributing and endorsing national organizations played a major role in the success of this CPG project.

Identified barriers and challenges that require a pro-active intervention to address them as a part of planning for implementation include, but are not limited to, the following: (i) medication availability, access, and sustainability; (ii) dissemination of the adapted CPG; (iii) lack of awareness of the primary care regarding the updated evidence-based recommendations of ADHD; (iv) lack of seamless integration between different national healthcare sectors; and (v) poor transition from pediatric to adult healthcare services.

An overall decision support record for the ADHD CPG adaptation group (GAG) using the KSU-Modified-ADAPTE methodology is provided in Appendix, Table 5. Additional details of the CPG adaptation methodology is made available from the Saudi ADHD Society (Link: https://cpg.adhd.org.sa/development/).

## Discussion

The aim of this study was to adapt the international CPGs and their recommendations to the Saudi healthcare context for the comprehensive management of people with ADHD across all local healthcare sectors.

The iterative process of the ADHD CPG adaptation reveals the nature of intensive work and capacity building that was an integral component of this project, and the specialized expertise required for such a process irrespective of the clinical or methodological expertise. The long timeline observed was not unique to this CPG adaptation project and was reported in other local CPG adaptation projects as well [12].

The GAG did not experience a shorter timeline for this CPG adaptation project compared to the 2–3-year period often suggested for de-novo development of CPGs [28]. This could be possibly due to the fact that we did not conduct this CPG adaptation process continuously and the GAG expert team members had other primary engagements and were not fully dedicated to this project. Indeed, it was obvious that the CPG adaptation process requires a considerable time commitment.

Nevertheless, the adaptation of CPG recommendations is a good and valid alternative to de-novo developing a CPG for people with ADHD, especially given the lack of relevant local high-quality systematic reviews and randomized controlled trials.

A strength of this study is the use of the ‘KSU-Modified-ADAPTE’ method because it is clearly structured and easy to follow with a set of tools to support the process.

Another noted strength was the inclusion of a patient advocate in the GAG with major contributions and input to the finalized adapted CPG.

There are increasing initiatives and projects related to knowledge translation in general and CPGs in particular nationally and regionally [12, 30, 31]. The World Health Organization Regional Office of the Eastern Mediterranean promotes and supports all advances in the development, adaptation, and implementation of CPGs at the regional level [32]. Furthermore, ‘National guidelines’ are core components of the ‘Model of Care’ of the new Saudi Arabian National Healthcare Plan [33, 34]].

For the medication recommendations and monitoring for both children and adults, we have minimal concerns about stimulant abuse in the local population since medication prescription has strict regulations in the country and has a clear system for controlled drugs regulations.

Conducting the needs assessment, that had a significant impact, is by default part of the set-up phase of adaptation and this is consistent with the experience of the GAG, which coincides with published evidence. This is an essential prerequisite of such a CPG adaptation project to practically determine the expected workload, resources, expertise, and the need for dedicated leadership and project management.

## Conclusions

The ‘KSU-Modified-ADAPTE’ methodology for CPG adaptation is a rigorous, practical, and intensive tool that—along with the AGREE II instrument as a major component of the adaptation process—has been demonstrated to be particularly feasible for national CPG projects.

Our experience with this adaptation methodology provides useful insight into its utilization on a national level in Saudi Arabia, and further demonstrates its potential suitability for the Eastern Mediterranean region. Additional modifications to the adaptation process and tools as per the context are recommended and accepted [12, 17].

Participation of a large number of healthcare sectors through multi-disciplinary groups in the CPG adaptation process aims at increasing the future uptake of the recommendations of this CPG. We anticipate an increase in the level of collaboration and integration of ADHD-related healthcare services as a result of the adoption of this adapted CPG.

### Implications for practice

Availability of a national CPG is essential but not sufficient to guarantee ultimate standardization of patient healthcare. The degree of positive impact on people with ADHD will highly depend on the effectiveness of dissemination and implementation strategies in addition to other quality improvement and safety interventions.

### Future research

A formal cost-analysis is suggested to decide whether the process of CPG adaptation is cost-effective. Research evidence is required as well to determine the effectiveness of CPGI tools and strategies for ADHD, as well as effectiveness of the adapted CPG in the following areas: (i) early identification and referral of children and adults with ADHD, (ii) appropriate transition of care from child to adult healthcare services for ADHD, (iii) parent training programs, (iv) initiation of drug treatment with dose adjustment as indicated, (v) regular assessment of the response to medication, and (vi) annual review of drug treatment.

## Supplementary information

**Additional file 1.** AGREE Reporting Checklist

## Data Availability

The data that support the findings of this study has been made available in the tables, figures and appendices of this article in addition to reference [1]. Further details could be made available from the authors upon reasonable request to the corresponding authors and the Saudi ADHD Society.

## Appendix

See Tables 3, 4, 5.

**Table 3:** Guideline adaptation working plan

CPG Phase		Tasks	Assigned to	Corresponding modules	Timeline
***Set up phase***					
Meeting 1		*Prepare for the adaptation process* Decide on health topic area Assess feasibility of adaptation Identify needed resources Establish multidisciplinary panel (adaptation working group) Write/ submit protocol Identify endorsing body Discuss authorship and accountability Discuss dissemination and implementation	CPG Adaptation Group (GAG)	Preparation module	January 2017
***Adaptation phase***					
Meeting 2		Decide on terms of reference/consensus process Establish CPG inclusion/exclusion criteria Help identify key search terms Help identify key documents/ sources Capacity building for CPG adaptation methodology (KSU-Modified-ADAPTE)	GAG	Preparation module	February 2017
		*Define Health Questions* Refine topic area	GAG	Scope and purpose module	
Meetings3–5		*Search and Screen CPGs* Complete CPG Search Narrow list of CPGs (if needed) by inclusion/ exclusion criteria *Assess CPGs* Complete AGREE II appraisal Assess CPG currency Complete evaluations (literature search and evidence, consistency of evidence and conclusions, conclusions and recommendations) for all recommendations (optional) Prepare recommendations matrix Assess acceptability	GAG	Search and screen module Assessment Module	March 2017 April 2017 April 2017
***Finalization Phase***					
Meeting 6		*Decide & select* Review all data Decide on recommendations for adapted CPG	GAG	Decision and selection module	July 2017
Online communication		*Draft CPG report* Write first draft of CPG and/or report on process	GAG	Customization module	March 2018
Online Communication		Approve first draft by CGC-s (Clinical content and Methodology)	GAG		July 2018
		*External review* Send for external review and consultation (Clinical content and Methodology) Get formal endorsement	External review panel for clinical content and methodology	External review module	December 2018–February 2019
Meetings 7–10		Discuss feedback Review and consultation	GAG	Aftercare planning module	April–May 2019 December 2019
		*Plan for future review & update* Decide on update process	GAG		
Meeting 11		*Produce final CPG* Create final adapted CPG *Including Implementation tool(s) and Performance Measures*	GAG	Final production module	
***Implementation Phase***					
Online communication		Consider implementation issues and strategies based on the Implementation tools and performance measures	GAG		July 2020

**Table 4:** Assessment of currency of the selected, Source CPG, currency survey of the CPG developer

Source CPG: Attention deficit hyperactivity disorder: diagnosis and management. NICE guideline [NG87]. Published date: 14th of March 2018
1. Are you aware of any new evidence relevant to this CPG statement? No
2. Is there any new evidence to invalidate any of the recommendations comprising the CPG? No
3. Are there any plans to update the CPG in the near future? No
4. When the CPG was last updated? 13th of September 2019 What is the citation for the latest version? https://www.nice.org.uk/guidance/ng87

**Table 5:** Decision support record for the ADHD CPG adaptation group (GAG) using the KSU-Modified-ADAPTE methodology

Phase	Module	Step	Tool	Decision		Reason (if not utilized)
				Utilized	Not utilized	
***ONE: SET-UP***	1.1 Preparation	1	1	√		
			2	√		
		2		√		
		3		√		
		4		√		
		5	3	√		
			4	√		
			1	√		
		6	5	√		
***TWO: ADAPTATION***	2.1. Scope and purpose	7	6	√		
	2.2. Search and screen	8	2	√		
			7	√		
		9	8	√		
		10	9		√	Not utilized. The GAG decided to rely on inclusion/ exclusion criteria and PIPOH compatibility
			10		√	
	2.3. Assessment	11	9	√		
			10	√		
		12	11	√		
		13	12		√	Not utilized. The GAG decided to select and adopt all the recommendations from one Source CPG: NICE NG87
		14	13		√	The GAG decided to rely on Domain 3 scores of the AGREE II appraisal
			14		√	
		15	15		√	The GAG decided to rely on Domains 2 and 5 scores of the AGREE II appraisal
	2.4. Decision and selection	16		√		
		17		√		Decision making and selection (5 options). The GAG adopted the recommendations with their evidence
	2.5. Customization	18	16	√		
**THRE**E: ***FINALIZATION***	3.1. External review and acknowledgment module	19	17	√		
		20		√		
		21		√		
		22		√		
	3.2. Aftercare planning	23	18	√		
	3.3. Final production	24		√		

## Abbreviations

ADHD: Attention deficit hyperactivity disorder
AGREE: Appraisal of guidelines for research and evaluation
CPG: Clinical practice guideline
CPGI: Clinical practice guideline implementation
GRADE: Grading of recommendations: assessment, development, and evaluation
KSU: King Saud University
NICE: National Institute for Health and Care Excellence
